# Editorial for the Special Issue on Micro/Nanofabrication for Retinal Implants

**DOI:** 10.3390/mi11111005

**Published:** 2020-11-14

**Authors:** Maesoon Im

**Affiliations:** 1 Brain Science Institute, KIST (Korea Institute of Science and Technology), Seoul 02792, Korea; maesoon.im@kist.re.kr or maesoon.im@gmail.com; 2 Division of Bio-Medical Science & Technology, UST (University of Science & Technology), Seoul 02792, Korea

The retinal prosthetic community has witnessed tremendous technological advances during the last two decades since the emergence of pioneering work [1]. However, clinical outcomes indicate it still needs substantial engineering endeavors to achieve near-normal vision. The early stage of microelectronic retinal prosthetic research seemed quite promising, with the best visual acuity restored by Argus systems of Second Sight Medical Products being much improved from 20/2520 to 20/1260 for versions I and II, respectively [2]. Moreover, the Alpha-IMS of the Retinal Implant further enhanced the best restored acuity to 20/546 with a short time interval [3,4]. Unfortunately, however, this best record remained unbroken for nearly 10 years until the recent report of PRIMA from Pixium Vision [5]. It is worth noting that even the newly achieved record, 20/460 [5], is still far below the level of legal blindness. This clearly suggests that, to practically help the visually impaired people, retinal prosthetic research has far further to go. In particular, appropriate micro/nanofabrication technologies are critical for the successful microelectronic prostheses. Accordingly, this Special Issue of *Micromachines*, entitled “Micro/Nanofabrication for Retinal Implants”, publishes two reviews and five research articles, which recapitulate stimulation approaches and introduce new electrode structures/materials for retinal prosthetic research, respectively.

For future innovations in retinal prosthetics, comprehensive understanding of the current status is essential. In that sense, the review paper of Shim et al. [6] is timely. They have systematically compared a broad array of existing methodologies for retinal stimulation. As is well summarized with several tables and figures, microelectronic approaches clearly need much improvement in spatial resolution. Although optical stimulation methods seem to offer big advantages for high-resolution artificial vision, Shim et al. concluded that they are somewhat less immature than electrical stimulation methods due to remaining safety and technical challenges. They also compared other stimulation modalities such as ultrasonic, magnetic, and chemical stimulation, which are all at the early stage of development for retinal prosthetic application. Among those new technologies, ultrasound stimulation has been particularly outlined in the other review paper by Lo et al. [7]. In the thorough review, they summarized previous representative works which acoustically stimulated not only the retina but also the visual cortex. Compared to the conventional microelectronic approaches, ultrasonic stimulation may achieve higher spatial resolution in a non-invasive and safe manner, making it an attractive alternative to electronic stimulation. However, Lo et al. pointed out that there is a trade-off between stimulation efficiency and spatial resolution. Additionally, sophisticated transducer arrays are required to restore complex visual percepts. Moreover, the underlying mechanism of ultrasonic stimulation needs to be unraveled.

In microelectronic approaches, stimulating electrodes have long been in two-dimensional planar shapes which typically touch the epi-/sub-retinal surface. Recently, these planar electrodes have been challenged by three-dimensional (3D) electrodes in diverse shapes [6,8,9]. In this Special Issue as well, the two articles by Shire et al. and Seo et al. [10,11] reported 3D pillar electrode structures. Their 3D microelectrodes are expected to be more efficient in subretinal stimulation by delivering electric current closer to target retinal neurons. From the system perspective, a more charge-efficient electrode design is preferred to increase the battery life of portable retinal prosthetics [12]. In addition, Ha et al. demonstrated that even the substrate can go 3D for a more effective recording of retinal spiking activities [13]. Given the intrinsic shape of the retina, their hemispherical microelectrode array may play a role in ex-vivo experiments for not only retinal prosthetic but also fundamental neurophysiology studies.

For a long time, it has been pointed out that non-uniform gap between epi-retinal electrode and retinal tissue creates irregular stimulation thresholds [2]. Primarily because the retina has a spherical shell-like shape, conformal coverage is highly challenging. In addition, the radius of curvature would be slightly different across individuals. To address this issue, Zhou et al. [14] proposed a hydrogel/elastomer bilayer and modulated the curvature of bilayers depending on monomer concentration. Additionally, the use of hydrogel as a substrate may improve long-term reliability of prostheses since hydrogel is known to be highly biocompatible [14]. Thus, this technology may be appropriate for wide-field retinal prosthetic electrode array.

As a new stimulation electrode material that may enhance performance of retinal implants, carbon nanotubes (CNTs) have recently became attractive due to their excellent electrical and mechanical properties [15]. In our Special Issue, Watterson et al. [16] reported that the biocompatibility and the mechanical integrity of CNT electrodes can be increased by adding an Al layer underneath an Fe layer which was used as a catalyst for the CNT growth. Similar efforts would be essential to upgrade biocompatibility when new materials are considered for retinal prosthetic applications.

In addition to numerous fabrication issues, it is critical for substantially improved artificial vision to consider neurophysiological and medical aspects of microelectronic retinal implants [17]. For instance, artificial visual percepts are likely to be natural if electrically-evoked neural activities are close matches of visually-evoked responses arising in healthy retinas [18]. However, given the remarkable complexity of the retina, it seems extremely challenging to closely mimic physiological (i.e., natural) spiking patterns in each retinal ganglion cell (RGC). Moreover, electric stimulation is known to indiscriminately activate diverse types of RGCs. Luckily, recent studies demonstrated that optimal stimulation parameters (e.g., stimulation frequency, pulse duration, and/or stimulus amplitude) can more selectively activate certain types of RGCs [19,20,21], probably making the whole retinal response more natural. In addition to these software approaches, hardware approaches such as novel electrode structures/materials may further enhance the selectivity. A more clinical consideration is that the degenerate retina demonstrated decreased consistency of electrically-evoked responses [22]. Therefore, response reliability may need to be guaranteed by innovative hardware/software strategies. Lastly, it would be great to test whether newly developed retinal implants elicit complex spiking responses similar to those arising in natural viewing conditions [23].
